# *N*-(2-Hy­droxy­phen­yl)-4-methyl­benzene­sulfonamide

**DOI:** 10.1107/S1600536813033394

**Published:** 2013-12-14

**Authors:** Shaaban K. Mohamed, Mehmet Akkurt, Benson M. Kariuki, Ali M. Ali, Mustafa R. Albayati

**Affiliations:** a Chemistry and Environmental Division, Manchester Metropolitan University, Manchester M1 5GD, England; b Chemistry Department, Faculty of Science, Minia University, 61519 El-Minia, Egypt; c Department of Physics, Faculty of Sciences, Erciyes University, 38039 Kayseri, Turkey; d School of Chemistry, Cardiff University, Main Building, Park Place, Cardiff, CF10 3AT, Wales; e Department of Chemistry, Faculty of Science, Sohag University, 82524 Sohag, Egypt; f Kirkuk University, College of Science, Department of Chemistry, Kirkuk, Iraq

## Abstract

In the title compound, C_13_H_13_NO_3_S, the dihedral angle between the benzene rings is 64.15 (7)° and the C—S—N—C torsion angle is −57.18 (12)°. An intra­molecular N—H⋯O hydrogen bond closes an *S*(5) ring. In the crystal, O—H⋯O hydrogen bonds link the mol­ecules into *C*(8) chains propagating in [100]. Weak C—H⋯π inter­actions are also observed.

## Related literature   

For background to the biological activity of sulfonamide compounds, see: Ozbek *et al.* (2007 ▶); El-Sayed *et al.* (2011 ▶). For related structures, see: Gowda *et al.* (2008*a* ▶,*b* ▶,*c* ▶).
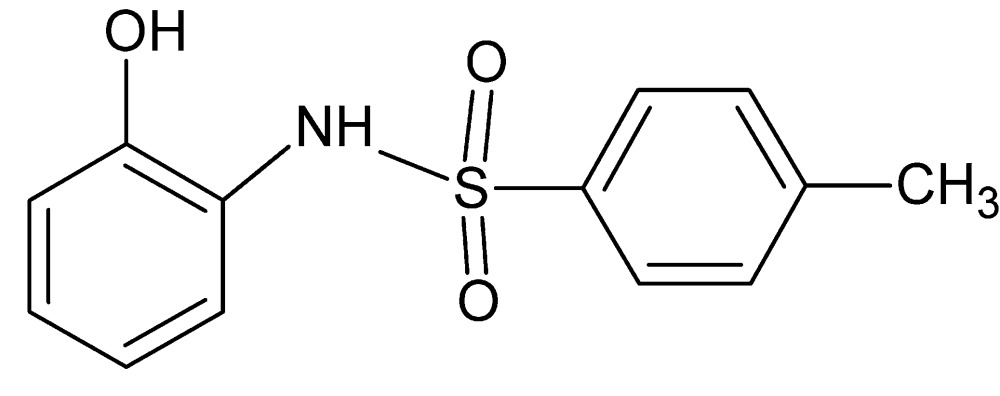


## Experimental   

### 

#### Crystal data  

C_13_H_13_NO_3_S
*M* *_r_* = 263.31
Monoclinic, 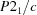

*a* = 7.6780 (1) Å
*b* = 15.4747 (3) Å
*c* = 10.7250 (2) Å
β = 104.333 (2)°
*V* = 1234.62 (4) Å^3^
*Z* = 4
Cu *K*α radiation
μ = 2.34 mm^−1^
*T* = 120 K
0.35 × 0.16 × 0.13 mm

#### Data collection  

Oxford Diffraction SuperNova (Dual, Cu at zero, Atlas) diffractometer
Absorption correction: multi-scan (*CrysAlis PRO*; Oxford Diffraction, 2013 ▶) *T*_min_ = 0.494, *T*_max_ = 0.750
4355 measured reflections
2377 independent reflections
2248 reflections with *I* > 2σ(*I*)
*R*_int_ = 0.011

#### Refinement  

*R*[*F* ^2^ > 2σ(*F* ^2^)] = 0.030
*wR*(*F* ^2^) = 0.082
*S* = 1.06
2377 reflections
172 parameters
H atoms treated by a mixture of independent and constrained refinement
Δρ_max_ = 0.28 e Å^−3^
Δρ_min_ = −0.38 e Å^−3^

### 

Data collection: *CrysAlis PRO* (Oxford Diffraction, 2013 ▶); cell refinement: *CrysAlis PRO*; data reduction: *CrysAlis PRO*; program(s) used to solve structure: *SHELXS97* (Sheldrick, 2008 ▶); program(s) used to refine structure: *SHELXL97* (Sheldrick, 2008 ▶); molecular graphics: *ORTEP-3 for Windows* (Farrugia, 2012 ▶); software used to prepare material for publication: *WinGX* (Farrugia, 2012 ▶) and *PLATON* (Spek, 2009 ▶).

## Supplementary Material

Crystal structure: contains datablock(s) global, I. DOI: 10.1107/S1600536813033394/hb7173sup1.cif

Structure factors: contains datablock(s) I. DOI: 10.1107/S1600536813033394/hb7173Isup2.hkl

Supporting information file. DOI: 10.1107/S1600536813033394/hb7173Isup3.cml

Additional supporting information:  crystallographic information; 3D view; checkCIF report

## Figures and Tables

**Table 1:** Hydrogen-bond geometry (Å, °) *Cg*1 and *Cg*2 are the centroids of the C1–C6 and C8–C13 benzene rings, respectively.

*D*—H⋯*A*	*D*—H	H⋯*A*	*D*⋯*A*	*D*—H⋯*A*
N1—H1*N*⋯O3	0.83 (2)	2.22 (2)	2.6420 (16)	111.6 (17)
O3—H1*O*⋯O2^i^	0.86 (2)	1.94 (2)	2.7852 (15)	172 (2)
C3—H3⋯*Cg*2^ii^	0.95	2.92	3.8022 (16)	155
C7—H7*C*⋯*Cg*1^iii^	0.98	2.85	3.5937 (17)	134

Symmetry codes: (i) 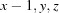; (ii) 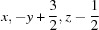; (iii) 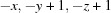.

**Fig. 1.**
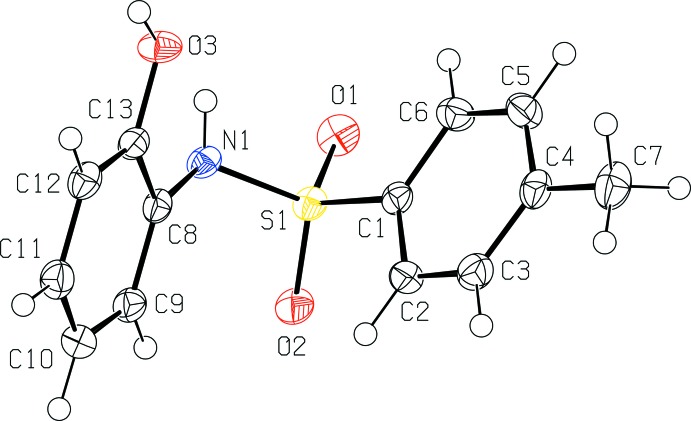
View of the title compound with displacement ellipsoids for non-H atoms drawn at the 50% probability level.

**Fig. 2.**
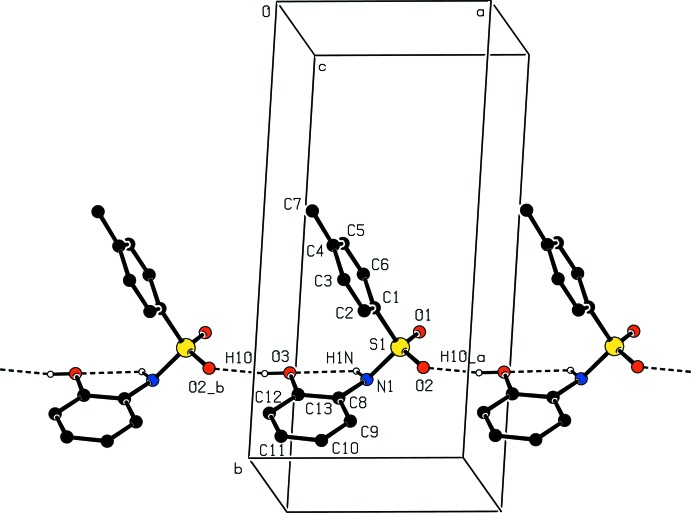
View of the hydrogen bonds along the a axis direction of the title compound. H bonds are shown as dashed lines.

**Fig. 3.**
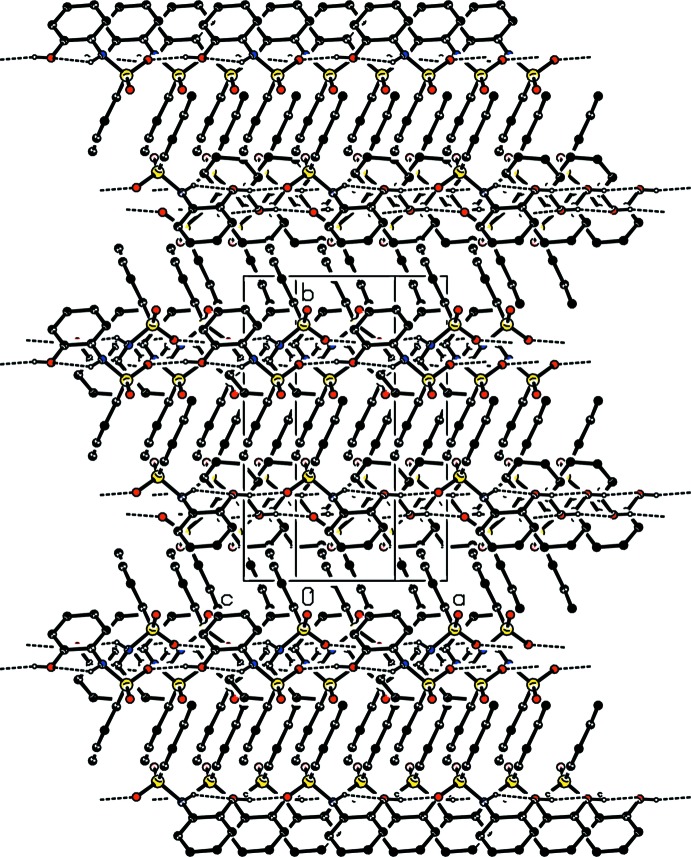
View of the molecular packing along the a axis of the title compound. H bonds are shown as dashed lines.

## supplementary crystallographic information

### 1. Comment

The biological activities of sulphonamide compounds are well documented, for example as antimicrobial (Ozbek *et al.*, 2007) and anticancer (El-Sayed *et al.*, 2011) agents. Further to our interest in related compounds with potential biactivity, we now report the synthesis and crystal structure of the title compound.

The benzene rings (C1–C6 and C8–C13) of the title compound (I) in Fig. 1 make a dihedral angle of 64.15 (7)° with each other. The bridge C1—S1—N1—C8 torsion angle between the benzene rings is -57.18 (12)°. The O1–S1–O2 and C1–S1–N1 angles are 119.78 (6) and 107.97 (6)°, respectively. The bond lengths and angles are similar to those in related structures (Gowda *et al.*, 2008**a*,*b*,c*).

The molecular conformation features an N—H···O hydrogen bond which forms an S(5) ring (Fig. 2). In the crystal, molecules are linked by O—H···O hydrogen bonds into C(8) chains along [100] (Figs. 2 and 3). Weak C—H···π interactions are also observed (Table 1).

### 2. Experimental

A mixture of 2-aminophenol (109 mg, 1 mmol) and *p*-toluenesulfonyl chloride (190 mg, 1 mmol) in 10 ml dioxane with addition of few drops of triethylamine as a catalyst, was refluxed for 4 h. The reaction mixture was left to cool at ambient temperature where the solid product was deposited, collected by filteration and recrystallized from ethanol in 91% yield. Brown needles were grown from ethanol solution over 3 days at room temperature. *M*.p. 391 K.

### 3. Refinement

The H atoms of the NH and OH groups were found from difference Fourier maps and refined freely. The C-bound H atoms were positioned geometrically, with C—H = 0.95 and 0.98 Å and refined as riding with *U*_iso_(H) = 1.*U*_eq_(C) for the methyl H atoms and *U*_iso_(H) = 1.2*U*_eq_(C) for the other H atoms.

### Crystal data

**Table:**

C_13_H_13_NO_3_S	*F*(000) = 552
*M**_r_* = 263.31	*D*_x_ = 1.417 Mg m^−^^3^
Monoclinic, *P*2_1_/*c*	Cu *K*α radiation, λ = 1.54180 Å
Hall symbol: -P 2ybc	Cell parameters from 2248 reflections
*a* = 7.6780 (1) Å	θ = 5.1–73.2°
*b* = 15.4747 (3) Å	µ = 2.34 mm^−^^1^
*c* = 10.7250 (2) Å	*T* = 120 K
β = 104.333 (2)°	Needle, brown
*V* = 1234.62 (4) Å^3^	0.35 × 0.16 × 0.13 mm
*Z* = 4	

### Data collection

**Table:**

Oxford Diffraction SuperNova (Dual, Cu at zero, Atlas) diffractometer	2377 independent reflections
Radiation source: SuperNova (Cu) X-ray Source	2248 reflections with *I* > 2σ(*I*)
Mirror monochromator	*R*_int_ = 0.011
ω scans	θ_max_ = 73.2°, θ_min_ = 5.1°
Absorption correction: multi-scan (*CrysAlis PRO*; Oxford Diffraction, 2013)	*h* = −8→9
*T*_min_ = 0.494, *T*_max_ = 0.750	*k* = −19→12
4355 measured reflections	*l* = −11→13

### Refinement

**Table:**

Refinement on *F*^2^	0 restraints
Least-squares matrix: full	Hydrogen site location: mixed
*R*[*F*^2^ > 2σ(*F*^2^)] = 0.030	H atoms treated by a mixture of independent and constrained refinement
*wR*(*F*^2^) = 0.082	W = 1/[Σ^2^(*F*O^2^) + (0.0424*P*)^2^ + 0.5981*P*] where *P* = (*F*O^2^ + 2*F*C^2^)/3
*S* = 1.06	(Δ/σ)_max_ = 0.001
2377 reflections	Δρ_max_ = 0.28 e Å^−^^3^
172 parameters	Δρ_min_ = −0.38 e Å^−^^3^

### Special details

**Table:**

Geometry. Bond distances, angles *etc*. have been calculated using the rounded fractional coordinates. All su's are estimated from the variances of the (full) variance-covariance matrix. The cell e.s.d.'s are taken into account in the estimation of distances, angles and torsion angles
Refinement. Refinement on *F*^2^ for ALL reflections except those flagged by the user for potential systematic errors. Weighted *R*-factors *wR* and all goodnesses of fit *S* are based on *F*^2^, conventional *R*-factors *R* are based on *F*, with *F* set to zero for negative *F*^2^. The observed criterion of *F*^2^ > σ(*F*^2^) is used only for calculating -*R*-factor-obs *etc*. and is not relevant to the choice of reflections for refinement. *R*-factors based on *F*^2^ are statistically about twice as large as those based on *F*, and *R*-factors based on ALL data will be even larger.

### Fractional atomic coordinates and isotropic or equivalent isotropic displacement parameters (Å^2^)

**Table:**

	*x*	*y*	*z*	*U*_iso_*/*U*_eq_	
S1	0.51287 (4)	0.65871 (2)	0.83597 (3)	0.0203 (1)	
O1	0.57618 (13)	0.61174 (7)	0.95358 (10)	0.0281 (3)	
O2	0.63687 (13)	0.70958 (7)	0.78545 (10)	0.0267 (3)	
O3	0.00824 (14)	0.71635 (7)	0.81974 (10)	0.0264 (3)	
N1	0.36177 (15)	0.72621 (8)	0.86360 (11)	0.0208 (3)	
C1	0.40408 (17)	0.58685 (9)	0.71468 (13)	0.0196 (4)	
C2	0.3833 (2)	0.60851 (9)	0.58584 (14)	0.0239 (4)	
C3	0.2974 (2)	0.55124 (10)	0.49170 (14)	0.0268 (4)	
C4	0.23172 (19)	0.47243 (9)	0.52309 (14)	0.0247 (4)	
C5	0.2521 (2)	0.45305 (10)	0.65261 (15)	0.0278 (4)	
C6	0.3373 (2)	0.50922 (10)	0.74888 (14)	0.0253 (4)	
C7	0.1447 (2)	0.40915 (11)	0.42031 (17)	0.0343 (5)	
C8	0.25492 (18)	0.77847 (9)	0.76315 (13)	0.0192 (3)	
C9	0.3290 (2)	0.83739 (9)	0.69307 (15)	0.0243 (4)	
C10	0.2174 (2)	0.89020 (9)	0.60237 (15)	0.0273 (4)	
C11	0.0320 (2)	0.88327 (10)	0.58080 (14)	0.0265 (4)	
C12	−0.04291 (19)	0.82447 (10)	0.65038 (14)	0.0234 (4)	
C13	0.06818 (18)	0.77298 (9)	0.74268 (13)	0.0202 (3)	
H1N	0.301 (3)	0.7023 (13)	0.9076 (19)	0.035 (5)*	
H1O	−0.106 (3)	0.7191 (15)	0.807 (2)	0.051 (6)*	
H2	0.42760	0.66200	0.56290	0.0290*	
H3	0.28280	0.56590	0.40370	0.0320*	
H5	0.20630	0.39990	0.67550	0.0330*	
H6	0.35000	0.49500	0.83680	0.0300*	
H7A	0.22220	0.35830	0.42470	0.0510*	
H7B	0.12710	0.43650	0.33560	0.0510*	
H7C	0.02810	0.39140	0.43350	0.0510*	
H9	0.45580	0.84160	0.70710	0.0290*	
H10	0.26800	0.93100	0.55520	0.0330*	
H11	−0.04390	0.91890	0.51810	0.0320*	
H12	−0.16980	0.81950	0.63480	0.0280*	

### Atomic displacement parameters (Å^2^)

**Table:**

	*U*^11^	*U*^22^	*U*^33^	*U*^12^	*U*^13^	*U*^23^
S1	0.0117 (2)	0.0246 (2)	0.0243 (2)	0.0002 (1)	0.0036 (1)	−0.0013 (1)
O1	0.0198 (5)	0.0360 (6)	0.0254 (5)	0.0033 (4)	−0.0005 (4)	0.0019 (4)
O2	0.0140 (5)	0.0298 (5)	0.0380 (6)	−0.0027 (4)	0.0096 (4)	−0.0031 (4)
O3	0.0146 (5)	0.0354 (6)	0.0298 (5)	−0.0019 (4)	0.0068 (4)	0.0060 (4)
N1	0.0150 (5)	0.0253 (6)	0.0229 (6)	−0.0006 (5)	0.0065 (5)	−0.0022 (5)
C1	0.0154 (6)	0.0207 (6)	0.0233 (7)	0.0027 (5)	0.0059 (5)	−0.0003 (5)
C2	0.0258 (7)	0.0208 (7)	0.0269 (7)	0.0013 (5)	0.0101 (6)	0.0031 (5)
C3	0.0308 (8)	0.0275 (7)	0.0232 (7)	0.0035 (6)	0.0089 (6)	0.0008 (6)
C4	0.0197 (6)	0.0247 (7)	0.0302 (7)	0.0040 (6)	0.0071 (6)	−0.0042 (6)
C5	0.0280 (8)	0.0210 (7)	0.0359 (8)	−0.0025 (6)	0.0107 (6)	0.0019 (6)
C6	0.0264 (7)	0.0254 (7)	0.0247 (7)	0.0003 (6)	0.0074 (6)	0.0047 (6)
C7	0.0288 (8)	0.0334 (8)	0.0404 (9)	−0.0004 (7)	0.0081 (7)	−0.0130 (7)
C8	0.0171 (6)	0.0196 (6)	0.0211 (6)	−0.0004 (5)	0.0052 (5)	−0.0056 (5)
C9	0.0206 (7)	0.0230 (7)	0.0319 (7)	−0.0026 (5)	0.0112 (6)	−0.0047 (6)
C10	0.0317 (8)	0.0223 (7)	0.0313 (8)	−0.0011 (6)	0.0144 (6)	0.0009 (6)
C11	0.0292 (8)	0.0243 (7)	0.0260 (7)	0.0048 (6)	0.0069 (6)	0.0001 (6)
C12	0.0178 (6)	0.0268 (7)	0.0255 (7)	0.0018 (5)	0.0051 (5)	−0.0041 (6)
C13	0.0186 (6)	0.0213 (6)	0.0220 (6)	−0.0023 (5)	0.0076 (5)	−0.0050 (5)

### Geometric parameters (Å, º)

**Table:**

S1—O1	1.4325 (11)	C8—C9	1.390 (2)
S1—O2	1.4405 (11)	C9—C10	1.391 (2)
S1—N1	1.6417 (12)	C10—C11	1.389 (2)
S1—C1	1.7574 (14)	C11—C12	1.389 (2)
O3—C13	1.3606 (18)	C12—C13	1.387 (2)
O3—H1O	0.86 (2)	C2—H2	0.9500
N1—C8	1.4318 (18)	C3—H3	0.9500
N1—H1N	0.83 (2)	C5—H5	0.9500
C1—C6	1.391 (2)	C6—H6	0.9500
C1—C2	1.392 (2)	C7—H7A	0.9800
C2—C3	1.382 (2)	C7—H7B	0.9800
C3—C4	1.393 (2)	C7—H7C	0.9800
C4—C7	1.502 (2)	C9—H9	0.9500
C4—C5	1.392 (2)	C10—H10	0.9500
C5—C6	1.384 (2)	C11—H11	0.9500
C8—C13	1.398 (2)	C12—H12	0.9500
			
O1—S1—O2	119.78 (6)	C11—C12—C13	119.77 (14)
O1—S1—N1	105.36 (6)	O3—C13—C8	115.57 (12)
O1—S1—C1	109.04 (6)	O3—C13—C12	124.25 (13)
O2—S1—N1	106.42 (6)	C8—C13—C12	120.17 (13)
O2—S1—C1	107.74 (6)	C1—C2—H2	120.00
N1—S1—C1	107.97 (6)	C3—C2—H2	120.00
C13—O3—H1O	111.0 (15)	C2—C3—H3	119.00
S1—N1—C8	121.50 (9)	C4—C3—H3	119.00
C8—N1—H1N	112.5 (15)	C4—C5—H5	119.00
S1—N1—H1N	110.0 (15)	C6—C5—H5	119.00
S1—C1—C6	119.35 (11)	C1—C6—H6	121.00
S1—C1—C2	119.94 (11)	C5—C6—H6	121.00
C2—C1—C6	120.71 (13)	C4—C7—H7A	109.00
C1—C2—C3	119.17 (13)	C4—C7—H7B	109.00
C2—C3—C4	121.39 (14)	C4—C7—H7C	109.00
C5—C4—C7	120.78 (13)	H7A—C7—H7B	109.00
C3—C4—C5	118.17 (13)	H7A—C7—H7C	109.00
C3—C4—C7	121.03 (13)	H7B—C7—H7C	109.00
C4—C5—C6	121.67 (14)	C8—C9—H9	120.00
C1—C6—C5	118.87 (13)	C10—C9—H9	120.00
N1—C8—C9	122.85 (13)	C9—C10—H10	120.00
N1—C8—C13	117.26 (12)	C11—C10—H10	120.00
C9—C8—C13	119.73 (13)	C10—C11—H11	120.00
C8—C9—C10	119.99 (14)	C12—C11—H11	120.00
C9—C10—C11	119.98 (14)	C11—C12—H12	120.00
C10—C11—C12	120.33 (14)	C13—C12—H12	120.00
			
O1—S1—N1—C8	−173.59 (11)	C2—C3—C4—C7	−177.69 (15)
O2—S1—N1—C8	58.25 (12)	C2—C3—C4—C5	1.0 (2)
C1—S1—N1—C8	−57.18 (12)	C3—C4—C5—C6	−1.0 (2)
O1—S1—C1—C2	−158.83 (12)	C7—C4—C5—C6	177.79 (15)
O2—S1—C1—C2	−27.38 (14)	C4—C5—C6—C1	−0.1 (2)
N1—S1—C1—C2	87.19 (13)	N1—C8—C9—C10	−175.85 (13)
O1—S1—C1—C6	22.08 (14)	C13—C8—C9—C10	−0.4 (2)
O2—S1—C1—C6	153.53 (12)	N1—C8—C13—O3	−1.43 (18)
N1—S1—C1—C6	−91.91 (13)	N1—C8—C13—C12	177.40 (13)
S1—N1—C8—C9	−59.30 (17)	C9—C8—C13—O3	−177.10 (13)
S1—N1—C8—C13	125.18 (12)	C9—C8—C13—C12	1.7 (2)
S1—C1—C6—C5	−179.91 (12)	C8—C9—C10—C11	−0.8 (2)
S1—C1—C2—C3	−180.00 (12)	C9—C10—C11—C12	0.7 (2)
C6—C1—C2—C3	−0.9 (2)	C10—C11—C12—C13	0.6 (2)
C2—C1—C6—C5	1.0 (2)	C11—C12—C13—O3	176.92 (13)
C1—C2—C3—C4	−0.1 (2)	C11—C12—C13—C8	−1.8 (2)

### Hydrogen-bond geometry (Å, º)

Cg1 and Cg2 are the centroids of the C1–C6 and C8–C13 benzene rings, respectively.

**Table:**

*D*—H···*A*	*D*—H	H···*A*	*D*···*A*	*D*—H···*A*
N1—H1*N*···O3	0.83 (2)	2.22 (2)	2.6420 (16)	111.6 (17)
O3—H1*O*···O2^i^	0.86 (2)	1.94 (2)	2.7852 (15)	172 (2)
C9—H9···O2	0.95	2.50	3.0531 (18)	117
C3—H3···*Cg*2^ii^	0.95	2.92	3.8022 (16)	155
C7—H7*C*···*Cg*1^iii^	0.98	2.85	3.5937 (17)	134

Symmetry codes: (i) *x*−1, *y*, *z*; (ii) *x*, −*y*+3/2, *z*−1/2; (iii) −*x*, −*y*+1, −*z*+1.
